# Comparison of the efficacy and safety of misoprostol and a Cook cervical ripening double-balloon catheter, alone and in combination, for cervical ripening and induction of labor

**DOI:** 10.3389/fmed.2026.1881105

**Published:** 2026-08-11

**Authors:** Dong Xu, Yanlan Sun, Zhenjing Chi, Ai Zhao, Haoyu Zheng

**Affiliations:** Department of Obstetrics, The Affiliated Huaian No.1 People’s Hospital of Nanjing Medical University, Huaian, Jiangsu, China

**Keywords:** bishop score, Cook cervical ripening double-balloon catheter, cervical ripening, labor induction, misoprostol

## Abstract

**Aim:**

This retrospective cohort study evaluated the associations of misoprostol alone, a Cook cervical ripening double-balloon catheter alone, and their combination for cervical ripening and labor induction in women with an unfavorable cervix.

**Methods:**

A total of 200 women with singleton term pregnancies, Bishop score ≤6, who underwent labor induction (April 2023–April 2024) were assigned to oral misoprostol alone (*n* = 80), a Cook cervical ripening double-balloon catheter alone (*n* = 60), or sequential misoprostol followed by the Cook double-balloon catheter (*n* = 60). The retrospective, non-randomized design precludes causal inference. The primary outcome was the 24-h change in Bishop score. Secondary outcomes included induction-to-delivery interval, vaginal delivery rate, and adverse events. Multivariable regression and propensity score matching were used to adjust for confounding.

**Results:**

The combination group showed a greater Bishop score increase (mean difference vs. misoprostol: 1.9, 95% CI 1.5–2.3; vs. Cook cervical ripening double-balloon catheter: 2.6, 95% CI 2.2–3.0; both *p* < 0.001) and a shorter induction-to-delivery interval (median difference vs. misoprostol: −4.7 h, 95% CI -6.0 to −3.5; vs. Cook cervical ripening double-balloon catheter: −4.2 h, 95% CI -5.5 to −3.0). Combination therapy was associated with higher odds of vaginal delivery (vs. misoprostol: aOR 2.45, 95% CI 1.45–4.14; vs. Cook cervical ripening double-balloon catheter: aOR 2.89, 95% CI 1.67–5.01). No statistically significant differences in measured short-term adverse events were observed, but the study was not powered to detect rare safety outcomes (e.g., uterine rupture). The *p*-value for uterine hyperstimulation (0.074) reflects statistical uncertainty.

**Conclusion:**

In this retrospective cohort, the sequential use of oral misoprostol followed by a Cook cervical ripening double-balloon catheter with a Cook cervical ripening double-balloon catheter was associated with more favorable cervical ripening and labor induction outcomes compared with either method alone. Because of the observational design, causality cannot be inferred. These findings suggest that combination therapy may be a reasonable option for appropriately selected patients, but confirmation in randomized controlled trials is needed.

## Introduction

Induction of labor (IOL) belongs to one of the most commonly performed interventions in modern obstetrics, accounting for approximately 20–25% of all pregnancies in developed countries, with rates continuing to rise due to increasing prevalence of conditions such as hypertensive disorders, gestational diabetes, and post-term pregnancies (1). When the cervix is favorable (Bishop score ≥7), IOL is often straightforward and associated with outcomes similar to spontaneous labor. However, when cervical status is unfavorable (Bishop score <6), the risks of failed induction, prolonged labor, uterine hyperstimulation, fetal distress, and cesarean delivery increase significantly (2). Therefore, effective and safe cervical ripening—the process of softening, effacing, and dilating the cervix—is a critical determinant of successful labor induction.

Currently, two main categories of cervical ripening methods are used: pharmacological and mechanical (“Cervical Ripening in Pregnancy: ACOG Clinical Practice Guideline No. 9,” 2025). Among pharmacological agents, misoprostol, a synthetic prostaglandin E1 analogue, is widely applied due to its low cost, oral or vaginal administration, and dual effects of cervical softening and uterine contraction stimulation (3). Oral misoprostol at 25 μg every 4 h has been shown to be effective and has a lower risk of uterine hyperstimulation relative to vaginal formulations. However, monotherapy with misoprostol carries an inherent risk of tachysystole and hyperstimulation, which may lead to non-reassuring fetal heart rate patterns and emergency cesarean delivery (4). Moreover, in some women, particularly those with a very unfavorable cervix (Bishop score ≤3), misoprostol alone may result in a prolonged latent phase and increased need for oxytocin augmentation (5).

Mechanical methods, such as cervical ripening balloon catheters, promote cervical ripening through sustained mechanical dilation of the internal os, which triggers local prostaglandin release and directly effaces the cervix (6). Mechanical methods do not increase the risk of uterine hyperstimulation and are therefore preferred in cases where prostaglandins are contraindicated (e.g., prior cesarean section, asthma, glaucoma) (7). However, their effects on cervical softening are less pronounced compared to pharmacological agents, and they may be associated with patient discomfort, displacement of the Cook cervical ripening double-balloon catheter, and a longer interval from induction to active labor. Additionally, mechanical methods alone may not adequately address the biochemical components of cervical ripening, such as collagen breakdown and glycosaminoglycan remodeling (8).

In recent years, combining misoprostol with a Cook cervical ripening double-balloon catheter has been hypothesized to produce a synergistic effect: the pharmacological agent softens the cervix at the biochemical level, while mechanical dilation promotes effacement and dilatation (9). This combined approach theoretically addresses both the biochemical and biomechanical aspects of cervical ripening, potentially accelerating the induction process and improving delivery outcomes without significantly increasing adverse events (10). Several small observational studies and a few randomized trials have suggested that combined therapy shortens the induction-to-delivery interval and reduces cesarean section rates relative to either method alone (11). However, most prior studies compared combination therapy with a single method (usually misoprostol alone), while direct three-way comparisons including a mechanical monotherapy arm are scarce (12). Large, well-designed retrospective or prospective comparative studies are still required to clarify the relative efficacy and safety profile of combined therapy versus each monotherapy.

To address this knowledge gap, the current study sought to evaluate the clinical efficacy and safety of three cervical ripening strategies—oral misoprostol alone, a Cook cervical ripening double-balloon catheter alone, and sequential oral misoprostol followed by the Cook double-balloon catheter—in women with singleton term pregnancies and an unfavorable cervix (Bishop score ≤6). Through analysis of maternal and neonatal outcomes in a retrospective cohort of 200 cases, we aimed to offer evidence-based recommendations to guide clinical decisions when selecting the optimal cervical ripening approach in this frequently encountered obstetric situation.

## Methods

### Study design and ethics

This was a single-center retrospective cohort study of 200 women who underwent labor induction between April 2023 and April 2024 at our hospital. The study was approved by the Institutional Review Board. Because this study involved only retrospective analysis of de-identified clinical data collected during routine clinical care, and no additional interventions or patient contact were performed, the requirement for written informed consent was waived by the IRB. All data were anonymized prior to analysis.

### Study population and grouping

Patients were assigned to three groups according to the induction method:

Group M (*n* = 80): oral misoprostol alone.

Group B (*n* = 60): Cook cervical ripening double-balloon catheter alone.

Group MB (*n* = 60): sequential oral misoprostol followed by a Cook cervical ripening double-balloon catheter.

Inclusion criteria: (1) Singleton pregnancy at ≥37 weeks of gestation; (2) Cephalic presentation; (3) Bishop score ≤6; (4) Clear medical or obstetric indication for induction (e.g., hypertensive disorders, gestational diabetes, post-term pregnancy); (5) Intact or ruptured membranes without signs of infection.

Exclusion criteria: (1) Previous cesarean section or other uterine surgery; (2) Placenta previa, vasa previa, or unexplained antepartum bleeding; (3) Placental abruption; (4) Known allergy to prostaglandins; (5) Fetal malpresentation or major anomalies; (6) Suspected intrauterine infection.

### Data collection and definitions

Baseline demographic and clinical characteristics were extracted from electronic medical records. Membrane status was determined by sterile speculum examination prior to induction. Bishop score components were assessed by a senior obstetric resident or attending physician at the time of induction. Estimated fetal weight was based on ultrasound examination performed within 7 days before induction. Hypertensive disorders were classified according to the American College of Obstetricians and Gynecologists guidelines: chronic hypertension, gestational hypertension, preeclampsia without severe features, and preeclampsia with severe features. Gestational diabetes was categorized as diet-controlled or medication-controlled (insulin or oral hypoglycemic agents). Oxytocin augmentation was defined as administration of oxytocin after the initial induction method (misoprostol, Cook cervical ripening double-balloon catheter, or both) according to the protocol described above. All variables were collected prospectively into a dedicated induction database and analyzed retrospectively.

### Interventions

#### Group M (oral misoprostol alone)

Oral misoprostol was initiated at 25 μg. Uterine contractions and fetal heart rate were reassessed after 2 h. Subsequent doses of 25-50 μg were administered at 4-h intervals according to uterine activity and clinical assessment, with a maximum cumulative dose of 200 μg within 24 h. Before each dose, uterine contraction frequency and fetal heart rate were assessed via cardiotocography (CTG) for at least 20 min. If uterine tachysystole (≥6 contractions in 10 min for two consecutive 10-min windows) or hyperstimulation (tachysystole with fetal heart rate decelerations) was observed, the next dose was withheld, and the patient was repositioned and given intravenous fluids as needed. If contractions subsided within 30 min, misoprostol could be resumed at the same dose after a 2-h observation period; if hyperstimulation persisted, the regimen was discontinued. No dose adjustment based on Bishop score progression was performed. No oxytocin was administered concurrently with misoprostol during the first 24 h unless specifically indicated for labor augmentation after rupture of membranes. The actual number of misoprostol doses administered and cumulative dose were recorded.

#### Group B (Cook cervical ripening double-balloon catheter alone)

Under sterile speculum examination, an Cook catheter was inserted through the cervical os into the endocervical canal. The Cook cervical ripening double-balloon catheter was inflated with 80 mL of normal saline (initial volume 40 mL, increased stepwise by 80 mL if the patient tolerated without significant discomfort). The external end of the catheter was taped to the inner thigh with gentle traction (approximately 50–100 g tension maintained by a rubber band or adhesive tape). The Cook cervical ripening double-balloon catheter was left in place for up to 24 h or until spontaneous expulsion. Maternal vital signs, uterine activity, and fetal heart rate were monitored every 30 min for the first 2 h, then hourly. If the Cook cervical ripening double-balloon catheter was still in place at 24 h, it was removed regardless of cervical dilation. After removal, a repeat Bishop score was assessed, and if active labor had not commenced (cervical dilation <4 cm with regular contractions), oxytocin augmentation could be initiated according to the hospital protocol.

#### Group MB (combined misoprostol and Cook cervical ripening double-balloon catheter)

The combined regimen was initiated either simultaneously (take misoprostol orally for 12 hours, and then insert the balloon) or, in cases where Cook cervical ripening double-balloon catheter insertion caused significant discomfort, misoprostol was started within 30 min after Cook cervical ripening double-balloon catheter placement. Oral misoprostol was administered at the same fixed dose as Group M. The Cook cervical ripening double-balloon catheter was inserted and managed identically to Group B: it was left in place for up to 24 h or until spontaneous expulsion. CTG monitoring was performed continuously for the first 2 h after initiation of the combined regimen and then every 30 min during active labor; during the latent phase, intermittent monitoring every 30–60 min was allowed. If uterine hyperstimulation occurred, misoprostol was withheld immediately, but the Cook cervical ripening double-balloon catheter was left in place (unless there were signs of placental abruption or non-reassuring fetal status). After symptoms resolved, misoprostol could be resumed at a reduced dose (12.5 μg every 4 h) if cervical ripening remained insufficient. The number of misoprostol doses, cumulative misoprostol dose, and Cook cervical ripening double-balloon catheter dwell time were recorded for Group MB.

#### Oxytocin augmentation protocol (all groups)

Oxytocin was initiated if active labor had not begun after completion of the assigned cervical-ripening protocol or if cervical ripening remained inadequate. Oxytocin was administered for augmentation. Oxytocin was started at 2 mU/min and increased by 2 mU/min every 30 min until three contractions per 10 min were achieved, to a maximum of 20 mU/min. In In Group M, oxytocin was started at least 2 h after the last misoprostol dose. In Group B, it could be started after balloon removal. In Group MB, it could be started after completion of the 12-h misoprostol phase followed by balloon removal. In Group MB (combined therapy), oxytocin was started only if both methods had been used for at least 24 h and the balloon had been removed. Cardiotocography (CTG) was performed continuously during oxytocin infusion.

### Outcome measures

Primary outcome: The primary outcome was the change in Bishop score from baseline to 24 h after induction. The scoring system evaluates five cervical parameters, with point assignments as shown in the following Table. The total Bishop score ranges from 0 to 13, with higher scores indicating greater cervical maturity (13). A Bishop score ≥7 is generally considered indicative of a favorable cervix for labor induction (14).

Bishop score scoring criteria.

**Table:** 

Parameter	0 points	1 points	2 points	3 points
Cervical dilation (cm)	0	1–2	3–4	≥5
Cervical effacement (%)	0–30	40–50	60–70	≥80
Fetal station	−3	−2	−1, 0	+1, +2
Cervical consistency	Firm	Medium	Soft	—
Cervical position	Posterior	Mid	Anterior	—

Secondary outcomes included:

Proportion of patients achieving Bishop score ≥7 at 24 h (a standard threshold for a favorable cervix).

Induction-to-delivery interval (hours, from start of induction to delivery).

Time from induction to active labor (hours, active labor was initially defined as cervical dilation ≥4 cm with regular painful contractions, which was the institutional protocol during the study period). While contemporary guidelines (21) recommend a 6 cm threshold for spontaneous labor, many induction studies still use 4 cm as a pragmatic endpoint for assessing the latent phase. To ensure comparability with newer studies, we performed a sensitivity analysis using a 6 cm threshold.

Duration of first, second, and third stages of labor (hours or minutes).

Total labor duration (hours, from active labor to delivery).

Delivery mode – vaginal delivery (spontaneous or assisted) vs. cesarean section. For women who underwent cesarean delivery, the primary indication was abstracted from the medical record and classified according to standard definitions: Failed induction: failure to achieve active labor (cervical dilation ≥4 cm with regular contractions) after ≥24 h of oxytocin augmentation following cervical ripening. Arrest of dilation: no cervical change for ≥4 h after active labor onset (≥4 cm) with adequate uterine contractions (≥200 Montevideo units or ≥3 contractions per 10 min). Arrest of descent: no fetal descent (station change) for ≥2 h in the second stage (≥3 h if neuraxial anesthesia). Non-reassuring fetal heart rate (FHR): persistent category II or III FHR tracings that did not resolve with intrauterine resuscitation and prompted immediate delivery. Cephalopelvic disproportion (CPD): clinical diagnosis (poor labor progress with adequate contractions and suspected fetal-pelvic mismatch) or sonographic evidence (estimated fetal weight >4,000 g with maternal pelvic findings). Other: maternal request, severe preeclampsia/eclampsia, placental abruption, cord prolapse.

Postpartum blood loss (mL, measured by graduated collection bags and weighed drapes).

Neonatal outcomes: birth weight (g), 1-min and 5-min Apgar scores, Fetal distress: defined as persistent category II or III fetal heart rate tracings (according to the NICHD classification) that did not resolve with intrauterine resuscitation (maternal repositioning, intravenous fluids, oxygen, or tocolysis) and prompted operative delivery (cesarean or instrumental vaginal delivery). Neonatal asphyxia: defined as a 5-min Apgar score <7, consistent with ACOG/AAP criteria.

Adverse events: uterine hyperstimulation: defined as tachysystole (≥6 contractions in 10 min for two consecutive 10-min windows) associated with fetal heart rate decelerations (late decelerations or variable decelerations lasting ≥60 s), based on AWHONN criteria. Postpartum hemorrhage: defined as cumulative blood loss ≥500 mL for vaginal delivery or ≥1,000 mL for cesarean delivery within 24 h after delivery, measured using graduated collection bags and weighed surgical drapes (ACOG definition). Meconium-stained fluid: defined as the presence of meconium in the amniotic fluid noted at the time of membrane rupture (spontaneous or artificial).

Bishop scores were assessed at baseline (immediately before induction) and at 24 h after induction (or earlier if active labor commenced). All assessments were performed by senior obstetric residents (post-graduate year 4 or above) or attending physicians who had completed a standardized cervical examination training program as part of the hospital’s labor induction protocol. Examiners were not formally tested for interobserver reliability, as this was a retrospective study using routine clinical data. The assessments were recorded in the medical record as part of routine care, and all examiners were blinded to the study hypothesis.

### Statistical analysis

Analyses were performed using SPSS version 26.0 (IBM Corp., Armonk, NY, USA) and R version 4.2.0 (R Foundation for Statistical Computing, Vienna, Austria).

The Shapiro–Wilk test was used to assess normality of continuous variables, and Levene’s test was applied to check homogeneity of variances.

Normally distributed variables (maternal age, gestational age, BMI, baseline Bishop score, Bishop score at 24 h, postpartum blood loss, birth weight, Apgar scores) are reported as mean ± standard deviation (SD). For comparisons across the three groups, one-way analysis of variance (ANOVA) was employed. When ANOVA revealed a significant overall difference, *post hoc* pairwise comparisons were carried out using Bonferroni correction; adjusted *p*-values are reported. Effect sizes for normally distributed variables are presented as mean differences (MD) with 95% confidence intervals (CI).

Non-normally distributed variables (labor duration variables: time from induction to active labor, first stage duration, second stage duration, total labor duration, induction-to-delivery interval) were confirmed by Shapiro–Wilk test (*p* < 0.05 for all). These are reported as median (interquartile range, IQR). The Kruskal-Wallis *H* test was used for three-group comparisons, followed by Dunn’s test for pairwise comparisons with Bonferroni correction; adjusted p-values are reported. Effect sizes for non-normally distributed variables are presented as median differences (Hodges-Lehmann estimator) with 95% CI.

Categorical variables are expressed as frequencies (n) and percentages (%). Group comparisons were performed using the Pearson chi-square test or Fisher’s exact test when expected cell counts were below 5. For pairwise comparisons, Bonferroni correction was applied (*α* = 0.05/3 = 0.0167 for three pairwise comparisons). Unadjusted odds ratios (OR) with 95% CI are reported; where appropriate, adjusted OR (aOR) from multivariable models are also provided.

To adjust for potential confounding, multivariable logistic regression was performed for vaginal delivery. For uterine hyperstimulation, owing to the very small number of events (*n* = 9), adjusted logistic regression was not performed because of the risk of model instability and overfitting. Only unadjusted comparisons (Fisher’s exact test and unadjusted odds ratios) are reported, and these should be regarded as exploratory, with results reported as adjusted odds ratios (aOR) with 95% confidence intervals (CI). For induction-to-delivery interval, which showed a positive skew, multivariable linear regression was performed on log-transformed values, with results back-transformed to geometric mean ratios (GMR) and 95% CI. Additionally, Cox proportional hazards regression was used to model time from induction to vaginal delivery, with results expressed as adjusted hazard ratios (aHR) and 95% CI. For this analysis, time zero was the start of induction. Women who underwent cesarean delivery, those who had not delivered vaginally by 72 h after induction, or those with incomplete follow-up were censored at the time of cesarean delivery or at 72 h, whichever occurred first. Cesarean delivery was treated as a censoring event because the primary aim was to evaluate the association between induction method and the instantaneous risk (hazard) of vaginal delivery; the resulting cause-specific hazard ratio provides a valid measure of this association. The proportional hazards assumption was assessed using Schoenfeld residuals, globally and for each covariate, and no violations were detected (global *p* > 0.05 for all models).

All multivariable models adjusted for the following prespecified covariates: maternal age, BMI, parity (primiparous vs. multiparous), gestational age, baseline Bishop score, membrane status (intact vs. ruptured), induction indication (hypertensive disorders, gestational diabetes, post-term pregnancy, or other), and birth weight. Oxytocin augmentation was not included in the primary models because it is a post-intervention variable that may mediate the effect of the induction method; adjusting for it could introduce overadjustment bias.

To further reduce confounding, a propensity score for receiving combination therapy (Group MB) versus each monotherapy (Group M or Group B) was estimated using a logistic regression model including all baseline covariates listed above. One-to-one nearest-neighbor matching without replacement was performed using a caliper of 0.2 SD of the logit of the propensity score. Balance between matched groups was assessed using standardized mean differences (SMD), with SMD < 0.1 indicating good balance. After matching, the primary and secondary outcomes were reanalyzed using paired *t*-tests for normally distributed continuous outcomes (normality confirmed by Shapiro–Wilk test within matched samples), Wilcoxon signed-rank tests for non-normally distributed continuous outcomes (e.g., induction-to-delivery interval), and McNemar’s test for binary outcomes. For non-normally distributed outcomes, results are reported as median (IQR) with the corresponding Wilcoxon *p-*value; for normally distributed outcomes, mean ± SD with paired *t*-test *p-*value is reported.

To explore whether the association between induction method and outcomes varied by key baseline characteristics, prespecified subgroup analyses were performed based on (1) baseline Bishop score category (≤3 vs. 4–6) and (2) parity (nulliparous vs. multiparous). For each subgroup, the primary outcome (Bishop score change) and secondary outcomes (vaginal delivery, induction-to-delivery interval) were compared among the three groups using the same statistical methods as in the primary analysis (ANOVA or Kruskal-Wallis for continuous outcomes, chi-square for binary outcomes). To formally test for interaction, we added a treatment-by-subgroup interaction term in multivariable regression models (separately for Bishop score and parity) for the outcome of vaginal delivery. All subgroup analyses are considered exploratory due to reduced sample sizes and multiple comparisons; results should be interpreted cautiously.

All hypothesis tests were two-tailed, with *p* < 0.05 considered statistically significant. Effect sizes (Cohen’s d for continuous variables, Cramér’s V for categorical variables) are reported where relevant.

## Results

### Baseline characteristics

Baseline characteristics were largely comparable among the three groups (Table 1). Oxytocin augmentation rates differed significantly across groups (overall *p* = 0.019), being highest in the Cook cervical ripening double-balloon catheter alone group (70.0%) and lowest in the misoprostol alone group (47.5%). Membrane status, cervical dilation distribution, Bishop score components, estimated fetal weight, and indications for induction were similar across groups (all *p* > 0.05).

**Table 1:** Baseline characteristics of the three groups.

**Characteristic**	**Group M (*n* = 80)**	**Group B (*n* = 60)**	**Group MB (*n* = 60)**	***p*-value**
Maternal age (years)	29.2 ± 4.5	29.8 ± 4.0	29.3 ± 4.1	0.673
Gestational age (weeks)	39.0 ± 1.3	39.2 ± 1.1	39.1 ± 1.2	0.541
BMI (kg/m^2^)	28.4 ± 3.2	28.1 ± 3.0	28.3 ± 3.1	0.782
Primiparous, *n* (%)	50 (62.5)	36 (60.0)	35 (58.3)	0.892
Initial Bishop score (total)	3.5 ± 1.0	3.4 ± 1.1	3.6 ± 0.9	0.548
– Dilation (cm)	0.9 ± 0.4	0.8 ± 0.5	1.0 ± 0.4	0.421
– Effacement (%)	38.5 ± 12.3	36.9 ± 11.8	40.2 ± 13.1	0.382
– Station	−2.1 ± 0.6	−2.2 ± 0.5	−2.0 ± 0.6	0.294
– Consistency (firm = 0, med = 1, soft = 2)	0.5 ± 0.6	0.4 ± 0.5	0.6 ± 0.6	0.408
– Position (posterior = 0, mid = 1, ant = 2)	0.6 ± 0.5	0.5 ± 0.5	0.7 ± 0.5	0.357
Initial cervical dilation category, *n* (%)				0.682
– ≤1 cm	52 (65.0)	41 (68.3)	36 (60.0)	
– 1–2 cm	23 (28.8)	16 (26.7)	19 (31.7)	
– >2 cm	5 (6.3)	3 (5.0)	5 (8.3)	
Membrane status at induction, *n* (%)				0.805
– Intact	68 (85.0)	52 (86.7)	51 (85.0)	
– Ruptured	12 (15.0)	8 (13.3)	9 (15.0)	
Estimated fetal weight (g)	3,320 ± 410	3,290 ± 390	3,340 ± 420	0.603
Indication for induction				0.862
– Hypertensive disorders, n (%)	28 (35.0)	20 (33.3)	22 (36.7)	
Chronic hypertension	6 (7.5)	4 (6.7)	5 (8.3)	
Gestational hypertension	12 (15.0)	9 (15.0)	10 (16.7)	
Preeclampsia without severe features	7 (8.8)	5 (8.3)	5 (8.3)	
Preeclampsia with severe features	3 (3.8)	2 (3.3)	2 (3.3)	
– Gestational diabetes, n (%)	24 (30.0)	18 (30.0)	16 (26.7)	
Diet-controlled	18 (22.5)	13 (21.7)	12 (20.0)	
Medication-controlled	6 (7.5)	5 (8.3)	4 (6.7)	
– Post-term pregnancy (≥41 weeks)	16 (20.0)	14 (23.3)	13 (21.7)	
– Other (oligohydramnios, fetal concern)	12 (15.0)	8 (13.3)	9 (15.0)	
Received oxytocin, *n* (%)	38 (47.5)	42 (70.0)	32 (53.3)	0.019*
Time to oxytocin start (h)†	14.5 ± 4.2	10.2 ± 3.5	11.8 ± 3.9	<0.001
Cumulative misoprostol dose (μg)	105 ± 27.5	N/A	85 ± 25	0.089
Number of misoprostol doses	4.2 ± 1.1	N/A	3.4 ± 1.0	0.089
Balloon dwell time (hours)	N/A	10.5 ± 2.3	10.2 ± 2.5	0.512

† Calculated only among women who received oxytocin augmentation. *Statistically significant overall difference at *p* < 0.05.

In the misoprostol alone group, the mean cumulative misoprostol dose was 105 μg; in the combination group, it was 85 μg (*p* = 0.089). Mean Cook cervical ripening double-balloon catheter dwell time was similar between the balloon alone and combination groups (*p* = 0.512).

### Bishop score 24 h after induction

**Figure 1:**
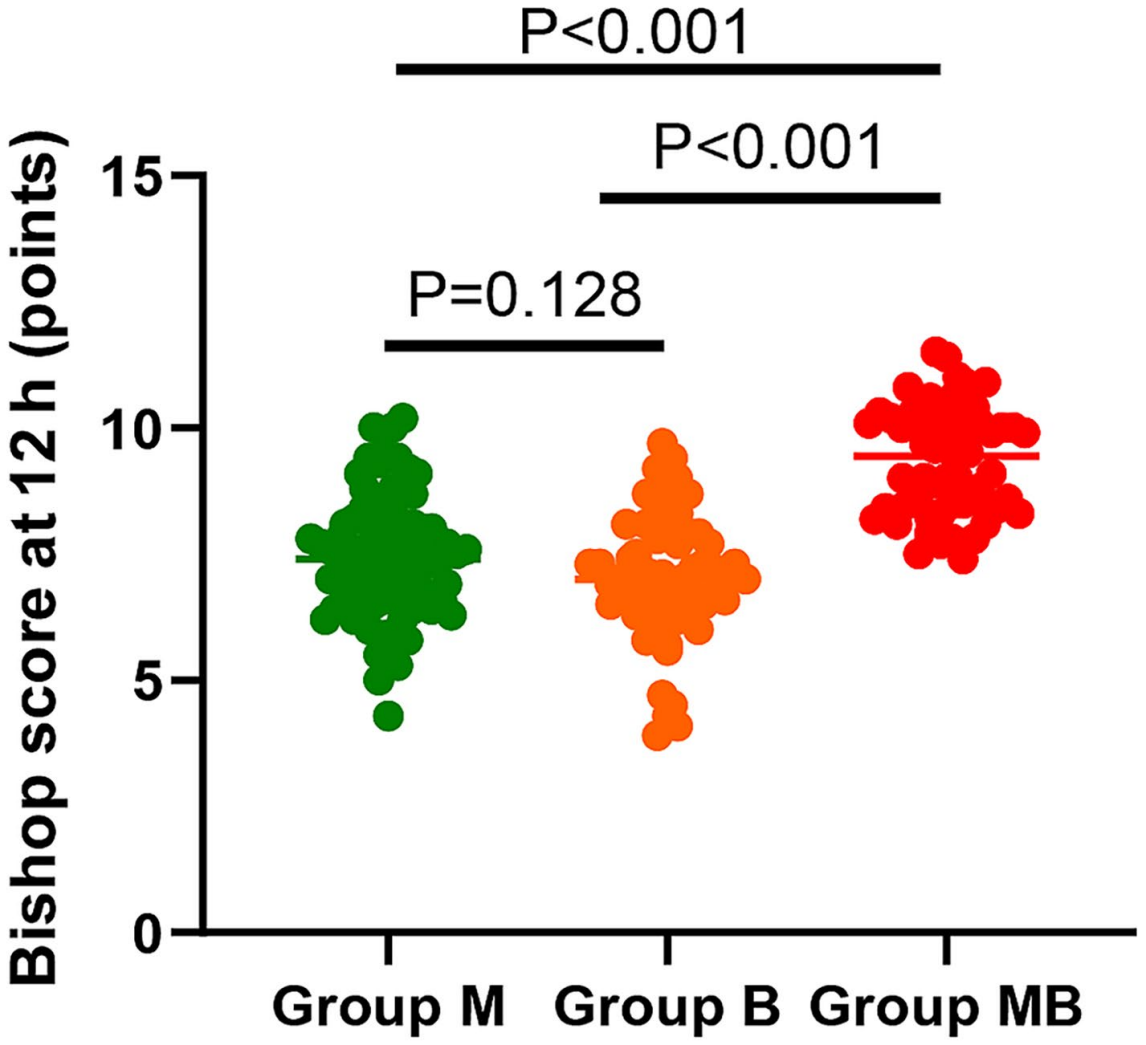
Bishop score 24 h after induction among the three groups.

The primary outcome (24-h change in Bishop score) showed a large overall difference (*p* < 0.001). The sequential strategy was associated with a significantly greater increase than misoprostol alone (mean difference 1.9, 95% CI 1.5-2.3) and the Cook cervical ripening double-balloon catheter alone (mean difference 2.6, 95% CI 2.2-3.0). The difference between the two monotherapies was not significant (*p* = 0.128). The proportion achieving a favorable cervix (Bishop score ≥7) was highest in the sequential-strategy group (70.0%) versus 48.8 and 36.7% (overall *p* = 0.001) (detailed data in Figure 1 and Table 2).

**Table 2:** Bishop score 24 h after induction of the three groups.

**Outcome**	**Group M (*n* = 80)**	**Group B (*n* = 60)**	**Group MB (*n* = 60)**	***p*-value**
Bishop score at 24 h	7.4 ± 1.3	6.6 ± 1.2	9.4 ± 1.1	<0.001
Bishop score increase from baseline	3.9 ± 1.1	3.2 ± 0.9	5.8 ± 1.2	<0.001
Bishop score ≥7 at 24 h, *n* (%)	39 (48.8)	22 (36.7)	42 (70.0)	0.001

### Labor duration

Labor duration outcomes (median, IQR) are summarized in Table 3. Combination therapy was associated with significantly shorter induction-to-active labor time (median differences: −4.1 h vs. misoprostol, −4.6 h vs. balloon) and shorter induction-to-delivery interval (median differences: −4.7 h vs. misoprostol, −4.2 h vs. balloon). First stage and total labor duration were also significantly shorter in the combination group, while second stage differences were not significant after correction for multiple testing (adjusted *p* = 0.089 for MB vs. B). Third stage did not differ across groups.

**Table 3:** Labor duration outcomes – median (IQR).

**Outcome**	**Group M (*n* = 80)**	**Group B (*n* = 60)**	**Group MB (*n* = 60)**	**Kruskal-Wallis *p***	**Pairwise (Bonferroni-adjusted *p*)**
Induction to active labor (h)	12.0 (10.0–14.5)	12.5 (10.2–15.0)	7.9 (6.5–9.8)	<0.001	MB vs. M: <0.001; MB vs. B: <0.001; M vs. B: 0.892
Induction to delivery (h)	18.5 (15.5–22.0)	18.0 (15.0–21.8)	13.8 (11.5–16.5)	<0.001	MB vs. M: <0.001; MB vs. B: <0.001; M vs. B: 0.756
First stage (h)	6.0 (4.5–7.5)	5.5 (4.2–7.0)	4.8 (3.5–6.0)	0.002	MB vs. M: 0.001; MB vs. B: 0.008; M vs. B: 0.234
Second stage (min)	55 (40–75)	50 (35–70)	45 (30–60)	0.013	MB vs. M: 0.006; MB vs. B: 0.089; M vs. B: 0.412
Total labor duration (h)	7.0 (5.5–8.8)	6.5 (5.0–8.2)	5.6 (4.2–7.0)	<0.001	MB vs. M: <0.001; MB vs. B: 0.014; M vs. B: 0.327
Third stage (min)	7 (5–10)	6 (5–9)	6 (5–8)	0.271	–

A sensitivity analysis using a 6 cm threshold for active labor (Table 4) confirmed the findings: combination therapy remained associated with shorter time to active labor (median differences: −4.3 h vs. misoprostol, −3.6 h vs. balloon; both *p* < 0.001).

**Table 4:** Time to active labor (6 cm threshold).

**Group**	**Median (IQR), hours**	**Pairwise median difference (95% CI)**	Adjusted *p*-value
Group M (*n* = 80)	18.5 (15.5–22.0)	Reference	–
Group B (*n* = 60)	17.8 (14.8–21.5)	−0.7 (−2.5 to 1.1)	0.58
Group MB (*n* = 60)	14.2 (11.5–17.0)	−4.3 (−5.8 to −2.9) vs. M; −3.6 (−5.2 to −2.1) vs. B	<0.001

### Delivery mode

The vaginal delivery rate was 86.7% in the combination group, compared with 71.3% in the misoprostol alone group and 66.7% in the balloon alone group (overall *p* = 0.018). After adjustment for confounders, combination therapy remained associated with higher odds of vaginal delivery (vs. misoprostol: aOR 2.45, 95% CI 1.45–4.14; vs. balloon: aOR 2.89, 95% CI 1.67–5.01). The lower cesarean rate in the combination group was primarily driven by fewer cesareans for failed induction (1.7% vs. 12.5 and 15.0%; overall *p* = 0.009) (Table 5).

**Table 5:** Indications for cesarean delivery by group.

**Indication**	**Group M (*n* = 80)**	**Group B (*n* = 60)**	**Group MB (*n* = 60)**	***p*-value**
Failed induction, *n* (%)	10 (12.5)	9 (15.0)	1 (1.7)	0.009
Arrest of dilation, *n* (%)	5 (6.3)	4 (6.7)	2 (3.3)	0.63
Arrest of descent, *n* (%)	3 (3.8)	3 (5.0)	1 (1.7)	0.59
Non-reassuring FHR, *n* (%)	5 (6.3)	5 (8.3)	3 (5.0)	0.78
Cephalopelvic disproportion, *n* (%)	2 (2.5)	1 (1.7)	1 (1.7)	0.92
Other, *n* (%)	1 (1.3)	1 (1.7)	0 (0)	0.75
Total cesarean deliveries, *n* (%)	23 (28.7)	20 (33.3)	8 (13.3)	0.018

### Time-to-event analysis

Cox proportional hazards regression showed that combination therapy was associated with a higher hazard of vaginal delivery compared to misoprostol alone (adjusted HR 1.92, 95% CI 1.52–2.43) and compared to balloon alone (adjusted HR 2.08, 95% CI 1.61–2.69), indicating a shorter time to vaginal delivery (Table 6). The proportional hazards assumption was satisfied for both comparisons (global Schoenfeld test *p* = 0.42 for MB vs. M; *p* = 0.38 for MB vs. B).

**Table 6:** Multivariable regression analyses for key outcomes.

**Outcome**	**Comparison**	**Adjusted effect estimate**	**95% CI**	***p*-value**
Vaginal delivery	MB vs. M	aOR 2.45	1.45–4.14	0.001
Vaginal delivery	MB vs. B	aOR 2.89	1.67–5.01	<0.001
Induction-to-delivery interval	MB vs. M	GMR 0.78	0.71–0.86	<0.001
Induction-to-delivery interval	MB vs. B	GMR 0.76	0.68–0.85	<0.001
Time to vaginal delivery	MB vs. M	aHR 1.92	1.52–2.43	<0.001
Time to vaginal delivery	MB vs. B	aHR 2.08	1.61–2.69	<0.001

All models adjusted for maternal age, BMI, parity, gestational age, baseline Bishop score, membrane status, induction indication, and birth weight. Owing to the low event count, no adjusted models were fitted for uterine hyperstimulation. CI, confidence interval; aOR, adjusted odds ratio; GMR, geometric mean ratio (induction-to-delivery interval modelled on log scale); aHR, adjusted hazard ratio. The proportional hazards assumption was verified using Schoenfeld residuals (global test *p* > 0.05 for all models). Cesarean deliveries and failure to deliver vaginally within 72 h were treated as censored.

### Postpartum blood loss and neonatal outcomes

**Figure 2:**
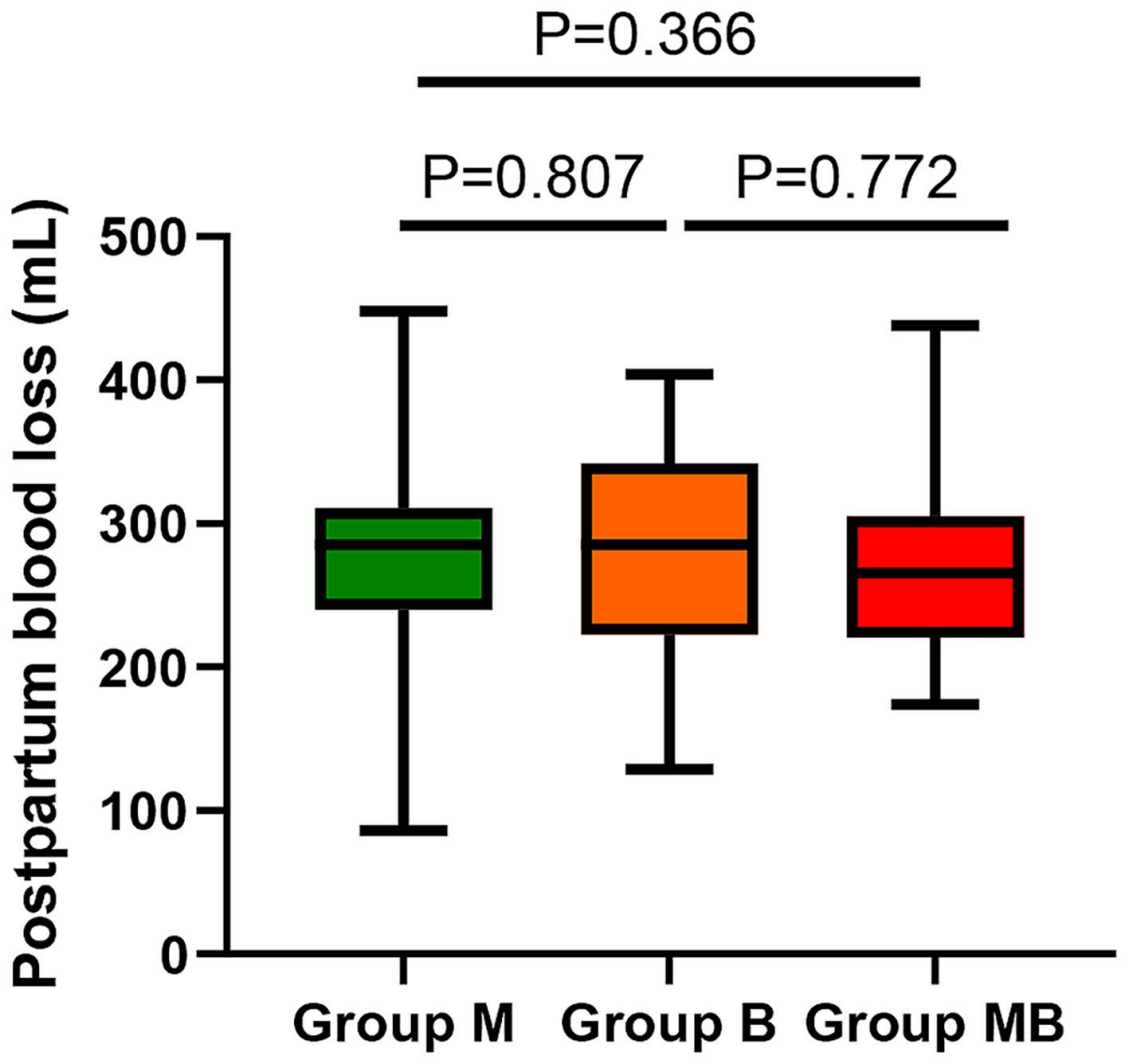
Postpartum blood loss among the three groups.

Postpartum blood loss did not differ significantly among groups (*p* = 0.807, *p* = 0.772, *p* = 0.366; Figure 2). Neonatal outcomes (birth weight, Apgar scores, fetal distress, neonatal asphyxia) were similar across groups, with no statistically significant differences (Table 5). Confidence intervals for rare events were wide (e.g., neonatal asphyxia: Group M 3.8% [95% CI 0.8–10.7%], Group MB 1.7% [95% CI 0.0–8.9%]), reflecting the limited sample size (Table 7).

**Table 7:** Neonatal outcomes of the three groups.

**Outcome**	**Group M (*n* = 80)**	**Group B (*n* = 60)**	**Group MB (*n* = 60)**	***p*-value**
Birth weight (g)	3,320 ± 420	3,280 ± 390	3,350 ± 410	0.612
1-min Apgar score	8.5 ± 0.8	8.6 ± 0.7	8.7 ± 0.6	0.253
5-min Apgar score	9.1 ± 0.4	9.2 ± 0.3	9.2 ± 0.3	0.184
Fetal distress, *n* (%)	6 (7.5)	5 (8.3)	3 (5.0)	0.751
Neonatal asphyxia, *n* (%)	3 (3.8)	2 (3.3)	1 (1.7)	0.755

### Adverse reactions

Uterine hyperstimulation occurred in 8.8% of the misoprostol alone group, 0% of the balloon alone group, and 3.3% of the sequential-strategy group (Fisher’s exact *p* = 0.074) (Table 8). Given the small number of events, no adjusted analyses were conducted, and the wide, overlapping confidence intervals (e.g., unadjusted OR for MB vs. M: 0.36, 95% CI 0.07–1.78) preclude any firm conclusions. These findings must be interpreted with extreme caution.

**Table 8:** Adverse reactions of the three groups.

**Outcome**	**Group M (*n* = 80)**	**Group B (*n* = 60)**	**Group MB (*n* = 60)**	**Overall *p***	**OR (95% CI) for MB vs M**	**OR (95% CI) for MB vs B**
Uterine hyperstimulation, *n* (%)	7 (8.8)	0 (0)	2 (3.3)	0.074	0.36 (0.07–1.78)	Not estimable
Postpartum hemorrhage (≥500 mL), *n* (%)	2 (2.5)	1 (1.7)	1 (1.7)	0.915	0.66 (0.06–7.43)	1.00 (0.06–16.4)
Meconium-stained fluid, *n* (%)	8 (10.0)	5 (8.3)	7 (11.7)	0.836	1.19 (0.41–3.47)	1.46 (0.44–4.87)

Uterine hyperstimulation: OR not estimable for MB vs. B because of zero events in the balloon group. Fisher’s exact test for the overall three-group comparison was *p* = 0.074. Given the rarity of events, all safety outcome estimates should be viewed as exploratory. CI, confidence interval; OR, odds ratio.

### Propensity score-matched analyses

Propensity score matching yielded well-balanced groups for both comparisons. Before matching, several covariates showed imbalances (SMD > 0.1); after matching, all SMDs were <0.1, indicating satisfactory balance (Supplementary Table S1). After matching, combination therapy remained associated with higher vaginal delivery rates (MB vs. M: 84.0% vs. 70.0%, *p* = 0.028; MB vs. B: 85.7% vs. 67.9%, *p* = 0.041) and shorter induction-to-delivery intervals. Induction-to-delivery intervals in the matched cohorts were as follows: for MB vs. M, median (IQR) 14.3 (11.9–16.8) h vs. 18.8 (15.6–22.4) h, Wilcoxon *p* < 0.001; for MB vs. B, median (IQR) 14.2 (11.7–16.9) h vs. 18.2 (15.2–21.9) h, Wilcoxon *p* < 0.001 (Table 9). Safety outcomes did not differ significantly after matching. Safety outcomes did not differ significantly after matching (Table 9).

**Table 9:** Propensity score-matched comparisons.

**Matched comparison**	**Outcome**	**Combined therapy**	**Monotherapy**	***p*-value**
MB vs. M (*n* = 50 pairs)	Vaginal delivery, *n* (%)	42 (84.0)	35 (70.0)	0.028
	Induction-to-delivery interval (h), median (IQR)	14.3 (11.9–16.8)	18.8 (15.6–22.4)	<0.001
	Uterine hyperstimulation, *n* (%)	2 (4.0)	4 (8.0)	0.414
MB vs. B (*n* = 56 pairs)	Vaginal delivery, *n* (%)	48 (85.7)	38 (67.9)	0.041
	Induction-to-delivery interval (h), median (IQR)	14.2 (11.7–16.9)	18.2 (15.2–21.9)	<0.001
	Uterine hyperstimulation, n (%)	2 (3.6)	0 (0)	0.500

Induction-to-delivery interval was non-normally distributed; therefore, matched values are summarized as median (interquartile range) and compared using the paired Wilcoxon signed-rank test. The median differences (Hodges-Lehmann estimates) are: MB vs. M, −4.5 h (95% CI −6.0 to −3.1); MB vs. B, −4.0 h (95% CI −5.5 to −2.6).

### Subgroup analyses

Of the 200 women, 51 had a baseline Bishop score ≤3 (very unfavorable cervix) and 149 had a score of 4–6 (moderately unfavorable). In both subgroups, combination therapy was associated with greater Bishop score improvement, shorter induction-to-delivery intervals, and higher vaginal delivery rates compared with either monotherapy (Tables 10, 11). For example, in the very unfavorable cervix subgroup (≤3), the vaginal delivery rate was 81.8% (9/11) in the combination group versus 50.0% (12/24) in the misoprostol alone group and 50.0% (8/16) in the balloon alone group (*p* = 0.045). In the moderately unfavorable cervix subgroup (4–6), the vaginal delivery rate was 87.8% (43/49) in the combination group versus 80.4% (45/56) in the misoprostol alone group and 72.7% (32/44) in the balloon alone group (*p* = 0.034). Bishop score improvement and induction-to-delivery intervals followed similar patterns.

**Table 10:** Outcomes by baseline Bishop score category–very unfavorable cervix (Bishop score ≤3).

**Outcome**	**Group M (*n* = 24)**	**Group B (*n* = 16)**	**Group MB (*n* = 11)**	***p*-value**
Bishop score increase (mean ± SD)	3.2 ± 0.9	2.8 ± 0.8	5.4 ± 1.1	<0.001
Vaginal delivery, *n* (%)	12 (50.0)	8 (50.0)	9 (81.8)	0.045
Induction-to-delivery interval (h), median (IQR)	20.5 (17.0–24.0)	19.5 (16.2–23.0)	15.0 (12.5–17.5)	<0.001

**Table 11:** Outcomes by baseline Bishop score category–moderately unfavorable cervix (Bishop score 4–6).

**Outcome**	**Group M (*n* = 56)**	**Group B (*n* = 44)**	**Group MB (*n* = 49)**	***p*-value**
Bishop score increase (mean ± SD)	4.2 ± 1.0	3.5 ± 0.9	6.0 ± 1.2	<0.001
Vaginal delivery, *n* (%)	45 (80.4)	32 (72.7)	43 (87.8)	0.034
Induction-to-delivery interval (h), median (IQR)	18.0 (15.0–21.0)	17.5 (14.5–20.5)	13.5 (11.0–16.0)	<0.001

Among nulliparous women (*n* = 121), combination therapy was associated with a vaginal delivery rate of 82.9% (29/35) compared with 64.0% (32/50) for misoprostol alone and 58.3% (21/36) for balloon alone (overall *p* = 0.045). Among multiparous women (*n* = 79), vaginal delivery rates were 92.0% (23/25) for combination therapy, 86.7% (26/30) for misoprostol alone, and 79.2% (19/24) for balloon alone (overall *p* = 0.39). The *p*-value for interaction was 0.12 (Tables 12, 13). The smaller number of multiparous women limited statistical power for this subgroup.

**Table 12:** Outcomes by parity–nulliparous women.

**Outcome**	**Group M (*n* = 50)**	**Group B (*n* = 36)**	**Group MB (*n* = 35)**	***p*-value**
Bishop score increase (mean ± SD)	3.6 ± 1.0	3.0 ± 0.9	5.6 ± 1.1	<0.001
Vaginal delivery, *n* (%)	32 (64.0)	21 (58.3)	29 (82.9)	0.045
Induction-to-delivery interval (h), median (IQR)	19.5 (16.5–23.0)	19.0 (15.8–22.5)	14.0 (11.5–17.0)	<0.001

**Table 13:** Outcomes by parity–multiparous women.

**Outcome**	**Group M (*n* = 30)**	**Group B (*n* = 24)**	**Group MB (*n* = 25)**	***p*-value**
Bishop score increase (mean ± SD)	4.4 ± 1.1	3.5 ± 0.9	6.1 ± 1.2	<0.001
Vaginal delivery, *n* (%)	26 (86.7)	19 (79.2)	23 (92.0)	0.39
Induction-to-delivery interval (h), median (IQR)	17.0 (14.5–20.0)	16.5 (13.8–19.5)	13.0 (11.0–15.5)	<0.001

## Discussion

In this retrospective cohort study, we found that sequential oral misoprostol followed by a Cook cervical ripening double-balloon catheter was associated with significantly improved cervical ripening outcomes compared to either agent alone. For the primary outcome (change in Bishop score from baseline to 24 h), the sequential-strategy group achieved a greater increase (5.8 ± 1.2) than misoprostol alone (3.9 ± 1.1) or Cook cervical ripening double-balloon catheter alone (3.2 ± 0.9). The proportion of women achieving a favorable cervix (Bishop score ≥7) was also highest in the sequential-strategy group (70.0% vs. 48.8 and 36.7%). In addition, the sequential strategy was associated with shorter induction-to-active labor time, shorter total labor duration, a shorter induction-to-delivery interval, and a higher vaginal delivery rate, without a statistically significant difference in the measured short-term adverse outcomes. Cox proportional hazards regression showed that the sequential strategy was associated with a higher hazard of vaginal delivery compared to misoprostol alone (adjusted HR 1.92, 95% CI 1.52–2.43) and compared to Cook cervical ripening double-balloon catheter alone (adjusted HR 2.08, 95% CI 1.61–2.69). Conversely, monotherapy with misoprostol alone and Cook cervical ripening double-balloon catheter alone produced comparable outcomes across most efficacy measures, suggesting that neither single method was clearly associated with superior efficacy in this population.

These findings align with and extend the existing literature on combination therapy for cervical ripening. Multiple randomized controlled trials and systematic reviews have demonstrated that the concurrent use of pharmacological and mechanical methods is associated with a reduced induction-to-delivery interval and a lower likelihood of cesarean section compared with misoprostol alone. For example, a meta-analysis by Ramos et al., which synthesized data from 11 systematic reviews encompassing 207 randomized trials, identified that combining a single-Cook cervical ripening double-balloon catheter catheter with misoprostol was the most effective approach for lowering the odds of cesarean delivery and minimizing prolonged time to vaginal birth (15). Likewise, a randomized trial by Soni et al. reported that in nulliparous women, the simultaneous application of a high-volume Foley catheter and vaginal misoprostol was associated with significantly shorter time from induction to delivery and the overall duration of labor (16). Nevertheless, the majority of earlier studies have primarily compared combination therapy with a single pharmacological agent, and relatively few have included a mechanical-only comparison group. Our study provides a direct three-way comparison, demonstrating that while combination therapy was associated with better outcomes than either monotherapy, misoprostol alone and Cook cervical ripening double-balloon catheter alone yielded similar efficacy. This finding is clinically relevant, as it suggests that when combination therapy is not feasible or contraindicated, either method may be chosen based on patient-specific factors (e.g., contraindications to prostaglandins) without a major apparent trade-off in efficacy as measured in this cohort.

The lower overall cesarean rate in the combination group (13.3%) compared to misoprostol alone (28.7%) and Cook cervical ripening double-balloon catheter alone (33.3%) was driven primarily by a marked reduction in cesareans for failed induction (1.7% vs. 12.5 and 15.0%, respectively). This pattern supports the clinical plausibility that more efficient cervical ripening with combination therapy was associated with a reduced risk of failed induction, rather than simply avoiding cesareans for other indications such as non-reassuring FHR, which occurred at similar rates across groups.

While combination therapy was associated with shorter induction-to-delivery intervals and higher vaginal delivery rates in our cohort, potential tradeoffs must be considered. Some recent large trials and meta-analyses have raised concerns that misoprostol-based combination regimens may be associated with increased maternal morbidity, particularly uterine hyperstimulation, intrapartum fever, and postpartum atony. For example, the PROBAAT-II trial (17) compared Foley catheter plus oral misoprostol with Foley catheter alone and found higher rates of uterine hyperstimulation with the combination (9% vs. 4%), although the difference was not statistically significant. A network meta-analysis by Ramos et al. (15) reported that while misoprostol plus Cook cervical ripening double-balloon catheter was the most effective strategy for achieving vaginal delivery, it was also associated with a higher risk of tachysystole compared with Cook cervical ripening double-balloon catheter alone. Similarly, a Cochrane review (6) noted that adding prostaglandins to mechanical methods may increase the risk of uterine hyperstimulation with fetal heart rate changes (RR 2.16, 95% CI 1.14–4.10).

In our study, uterine hyperstimulation rates were numerically lower in the combination group (3.3%) than in the misoprostol alone group (8.8%), with no events in the balloon group. However, the total number of events was very small (*n* = 9), and adjusted regression models were not performed due to the risk of statistical instability. The unadjusted odds ratio for MB vs. M was 0.36 (95% CI 0.07–1.78), with a Fisher’s exact *p* value of 0.074, indicating substantial uncertainty. These data should be viewed as exploratory and do not provide reliable evidence for or against a difference in hyperstimulation risk; definitive safety comparisons require far larger sample sizes.

Regarding neonatal safety, our study found no significant differences in Apgar scores, fetal distress, or neonatal asphyxia. This is consistent with most large trials, which have not demonstrated increased neonatal morbidity with misoprostol-based regimens when used at low doses (25 μg every 4 h). However, rare outcomes such as neonatal encephalopathy or NICU admission require much larger sample sizes to evaluate.

Comparison with recent Cook cervical ripening double-balloon catheter-based induction protocols: Our results align with the PROBAAT-II trial and the Finnish randomized pilot trial (18) in showing that combination therapy reduces induction-to-delivery time and cesarean rates compared with Cook cervical ripening double-balloon catheter alone. However, unlike the PROBAAT-II trial which used vaginal misoprostol (50 μg), our study used oral misoprostol (25 μg every 4 h), which may have a more favorable safety profile. Direct comparisons are limited by differences in dosing and administration routes. Our finding that Cook cervical ripening double-balloon catheter alone and misoprostol alone have comparable efficacy is novel and suggests that either monotherapy is a reasonable alternative when combination therapy is not feasible.

The observed associations are consistent with a potential synergistic effect of combination therapy, but the underlying mechanisms were not directly tested. Biologically, misoprostol softens the cervix biochemically while the Foley balloon provides mechanical dilation (19). It is hypothesized that these complementary actions may facilitate more efficient cervical ripening (20). However, this interpretation remains speculative, and causality cannot be inferred from this observational study.

No statistically significant differences in neonatal outcomes (Apgar scores, fetal distress, neonatal asphyxia) were observed across the three groups. However, the confidence intervals for these rare events were wide (e.g., for neonatal asphyxia: Group M 3.8% [95% CI 0.8–10.7%], Group MB 1.7% [95% CI 0.0–8.9%]), reflecting the limited sample size. The absence of a statistically significant difference does not constitute evidence of safety equivalence, and the study was not powered to detect rare but serious adverse events such as uterine rupture (none occurred), severe postpartum hemorrhage, or NICU admission.

Regarding uterine hyperstimulation, the observed rates were 8.8% (95% CI 3.6–17.2%) in Group M and 3.3% (95% CI 0.4–11.5%) in Group MB, with a *p*-value of 0.074. This *p*-value indicates statistical uncertainty rather than evidence of no difference. The wide confidence intervals overlap considerably, and a true difference in hyperstimulation risk cannot be ruled out. Given the small sample size and overlapping CIs, the numerical difference should not be overinterpreted, and no conclusions about mechanisms can be drawn from these data.

Postpartum blood loss was similar across groups, which is consistent with the absence of significant differences in uterine atony risk factors. The third stage of labor was also comparable, suggesting that neither the pharmacological nor mechanical methods had residual effects on placental separation or uterine contractility postpartum that were detectable in this study.

Exploratory subgroup analyses suggested that the benefits associated with combination therapy might be more pronounced in women with a very unfavorable cervix (Bishop score ≤3) and in nulliparous women, although interaction tests were not statistically significant. These findings are consistent with the hypothesis that women with the least favorable cervical conditions and those with no prior vaginal delivery may derive the greatest relative benefit from combined pharmacological and mechanical ripening. However, given the small sample sizes in some subgroups (e.g., only 11 women with Bishop score ≤3 received combination therapy) and the exploratory nature of these analyses, the results should be interpreted with caution and require confirmation in larger, adequately powered trials.

### Study strengths and limitations

This study has several strengths. First, it provides a direct three-way comparison of two widely used cervical ripening methods and their combination, addressing a gap in the literature where mechanical monotherapy is often underrepresented. Second, the use of oral misoprostol rather than vaginal formulations enhances generalizability to settings where oral administration is preferred. Third, the inclusion of detailed labor duration outcomes and cesarean indication analysis allows for a nuanced understanding of where time savings and delivery mode differences occurred. Fourth, the standardized protocols for oxytocin augmentation and management of hyperstimulation increase reproducibility. Fifth, we employed rigorous statistical methods to address confounding—including multivariable logistic/linear regression, Cox proportional hazards modeling, and propensity score matching with balance assessment—which strengthens the internal validity of the observed associations.

Nonetheless, several important limitations warrant acknowledgment.

1. Study design and confounding: The retrospective, non-randomized design remains the most important limitation. Because of this design, no causal conclusions can be drawn from this study. Treatment allocation was not randomized, and despite extensive adjustment for measured confounders using multivariable regression and propensity score matching, residual confounding by unmeasured variables cannot be entirely excluded. Potential unmeasured confounders include physician preference for certain induction methods (e.g., choosing the sequential strategy for patients perceived to have the most unfavorable cervices), patient refusal of Cook cervical ripening double-balloon catheter placement due to discomfort, subtle differences in cervical examination technique among providers, socioeconomic status, health literacy, prior vaginal delivery details, and cervical length or consistency not fully captured by the Bishop score. Furthermore, confounding by indication is inherent to non-randomized comparative effectiveness studies; women who received the sequential strategy may have differed in ways we could not measure.
2. Sample size and power: The sample size (*n* = 200, with only 60–80 per group) is modest. While the study had adequate power to detect moderate-to-large differences in efficacy outcomes (e.g., Bishop score change, vaginal delivery rate), it was not designed or powered to evaluate rare safety outcomes. Events such as uterine rupture, severe postpartum hemorrhage requiring transfusion, NICU admission, or neonatal encephalopathy occur at rates of <1% in term inductions; detecting a difference in such rare events would require thousands of patients. Consequently, our safety findings should be considered exploratory. The absence of a statistically significant difference in the measured adverse outcomes does not imply that no clinically important difference exists. Statistical non-significance does not prove equivalence or non-inferiority; a formal non-inferiority trial with prespecified margins would be required to make such a claim. Readers should interpret the safety data with caution.
3. Single-center and generalizability: The single-center design limits generalizability to other settings with different patient demographics, induction protocols, or cervical assessment practices. The Cook cervical ripening double-balloon catheter-alone group had a smaller sample size (*n* = 60) than the misoprostol-alone group (*n* = 80), which could affect precision and statistical power for comparisons involving that group.
4. Variability in cervical assessment: Bishop score assessment, while performed by trained obstetric residents and attending physicians, may have inherent inter-observer variability. No formal reliability testing (e.g., intraclass correlation coefficient) was performed because this was a retrospective analysis of routine clinical data. This potential misclassification could bias the primary outcome toward or away from the null, though the magnitude of such bias is unknown. Prospective studies should include standardized examiner training and interobserver reliability testing.
5. Missing data on dose and protocol details: We did not systematically record cumulative misoprostol dose per patient beyond the mean values reported (which were derived from chart review). More importantly, we did not collect detailed data on contraction frequency (other than for hyperstimulation events), fetal heart rate patterns, or protocol discontinuation reasons. Therefore, the mechanism underlying the numerically lower hyperstimulation rate in the combination group remains speculative, as discussed above.
6. Patient-reported outcomes not assessed: This study did not collect subjective outcomes such as pain during balloon placement, pelvic pressure, overall discomfort, maternal anxiety, or satisfaction with the induction process. These outcomes are particularly relevant for mechanical methods like the Cook cervical ripening double-balloon catheter, which may cause significant discomfort in some women. Because of the retrospective design, these data were not available in the medical records. Future prospective studies, including randomized controlled trials of cervical ripening methods, should incorporate validated patient-reported outcome measures (e.g., visual analog scale for pain, childbirth satisfaction questionnaires) to provide a more complete safety and acceptability profile and to support patient-centered care.

Given these limitations, a large, well-designed, multicenter randomized controlled trial is needed to confirm whether the associations we observed reflect causal effects and to establish optimal dosing regimens. Future studies should evaluate whether carefully titrated increases in the single dose of oral misoprostol used as monotherapy can improve cervical-ripening efficiency without increasing uterine hyperstimulation or other maternal and neonatal adverse outcomes. Such trials should be adequately powered to evaluate both efficacy and rare safety outcomes, and should include patient-reported outcomes and long-term follow-up.

### Clinical implications

Our findings support consideration of sequential oral misoprostol followed by a Cook cervical ripening double-balloon catheter as an effective cervical-ripening strategy in appropriately selected patients, with no statistically significant increase in measured short-term adverse outcomes in this cohort. This sequential strategy may be particularly beneficial in settings where rapid cervical ripening is desirable (e.g., maternal medical conditions requiring timely delivery) or where resources for prolonged induction are limited. The comparable efficacy of misoprostol alone and Cook cervical ripening double-balloon catheter alone provides flexibility: in patients with contraindications to prostaglandins (e.g., prior cesarean section, asthma, glaucoma), Cook cervical ripening double-balloon catheter alone is a reasonable alternative; in settings where mechanical devices are unavailable or contraindicated, oral misoprostol alone remains a viable option.

## Conclusion

In this retrospective cohort of women with term singleton pregnancies and an unfavorable cervix, sequential oral misoprostol followed by a Cook cervical ripening double-balloon catheter was associated with more favorable cervical ripening and labor induction outcomes compared with either method alone. Specifically, combination therapy was linked to a greater improvement in Bishop score, shorter induction-to-delivery intervals, and a higher vaginal delivery rate after adjustment for measured confounders.

When combination therapy was not used, monotherapy with either oral misoprostol or a Cook cervical ripening double-balloon catheter alone appeared to have comparable associations with efficacy outcomes. These findings suggest that combination therapy may be a reasonable option for cervical ripening in appropriately selected patients, but they should be confirmed in adequately powered randomized controlled trials that are specifically designed to evaluate both efficacy and rare safety outcomes.

## Data Availability

The original contributions presented in the study are included in the article/Supplementary material, further inquiries can be directed to the corresponding author.
