# Supplementary material for: Comparison of the efficacy and safety of misoprostol and a Cook cervical ripening double-balloon catheter, alone and in combination, for cervical ripening and induction of labor
Source: Front Med (Lausanne). 2026 Aug 11;13:1881105. doi: 10.3389/fmed.2026.1881105 (PMC13503514; doi:10.3389/fmed.2026.1881105)
Supplement: Supplementary file 1 [file Table_1.docx]

Supplementary Table S1. Balance of baseline covariates before and after propensity score matching

| **Covariate** | **MB vs. M** | **MB vs. M** | **MB vs. M** | **MB vs. B** | **MB vs. B** | **MB vs. B** |
| --- | --- | --- | --- | --- | --- | --- |
|  | **Before matching SMD** | **After matching SMD** | **After matching mean/prop. (MB/M)** | **Before matching SMD** | **After matching SMD** | **After matching mean/prop. (MB/B)** |
| Maternal age (years) | 0.04 | 0.02 | 29.3/29.2 | 0.11 | 0.05 | 29.3/29.7 |
| BMI (kg/m²) | 0.02 | 0.03 | 28.3/28.4 | 0.06 | 0.04 | 28.3/28.1 |
| Primiparous (%) | 0.09 | 0.04 | 58.3/60.0 | 0.03 | 0.02 | 58.3/60.7 |
| Gestational age (weeks) | 0.08 | 0.05 | 39.1/39.0 | 0.09 | 0.06 | 39.1/39.3 |
| Baseline Bishop score | 0.10 | 0.03 | 3.6/3.5 | 0.20 | 0.07 | 3.6/3.5 |
| Membrane status (% intact) | 0.00 | 0.02 | 85.0/85.0 | 0.05 | 0.03 | 85.0/86.7 |
| Induction indication (%) |  |  |  |  |  |  |
| – Hypertensive disorders | 0.04 | 0.02 | 36.7/35.0 | 0.07 | 0.04 | 36.7/33.3 |
| – Gestational diabetes | 0.07 | 0.04 | 26.7/30.0 | 0.07 | 0.05 | 26.7/30.0 |
| – Post‑term pregnancy | 0.04 | 0.02 | 21.7/20.0 | 0.04 | 0.03 | 21.7/23.3 |
| – Other | 0.00 | 0.01 | 15.0/15.0 | 0.05 | 0.03 | 15.0/13.3 |
| Birth weight (g) | 0.05 | 0.04 | 3350/3320 | 0.15 | 0.06 | 3350/3300 |

SMD, standardized mean difference. All post‑matching SMDs <0.1 indicate good covariate balance. For the MB vs. M comparison, 50 matched pairs were analyzed; for MB vs. B, 56 matched pairs.
